# Lingual Thyroid Excision with Transoral Robotic Surgery

**DOI:** 10.1155/2015/548582

**Published:** 2015-05-07

**Authors:** Elif Ersoy Callıoglu, Kazım Bozdemir, Bulent Ulusoy, Tolga Oguzhan, M. Hakan Korkmaz

**Affiliations:** ^1^Department of Otorhinolaryngology, Ministry of Health Ataturk Training and Research Hospital, 06800 Ankara, Turkey; ^2^Department of Otorhinolaryngology, Yıldırım Beyazıt University Faculty of Medicine, 06800 Ankara, Turkey

## Abstract

Ectopic thyroid gland may be detected at any place between foramen caecaum and normal thyroid localization due to inadequacy of the embryological migration of the thyroid gland. It has a prevalence varying between 1/10.000 and 1/100000 in the community. Usually follow-up without treatment is preferred except for obstructive symptoms, bleeding, and suspicion of malignity. Main symptoms are dysphagia, dysphonia, bleeding, dyspnea, and obstructive sleep apnea. In symptomatic cases, the first described method in surgical treatment is open approach since it is a region difficult to have access to. However, this approach has an increased risk of morbidity and postoperative complications. Transoral robotic surgery, which is a minimally invasive surgical procedure, has advantages such as larger three-dimensional point of view and ease of manipulation due to robotic instruments. In this report, a case at the age of 49 who presented to our clinic with obstructive symptoms increasing within the last year and was found to have lingual thyroid and underwent excision of ectopic thyroid tissue by da Vinci surgical system is presented.

## 1. Introduction

Thyroid gland develops from median endodermal folds at the fourth week of embryogenesis as thyroid diverticulum at the base of pharynx. It descends into its pretracheal position via thyroglossal ductus until the 7th week [1]. As a consequence of inadequacy of embryological migration of thyroid gland, ectopic thyroid tissue may be present anywhere between foramen caecum and normal thyroid localization. Although its most common localization has been described as lingual region by Hickman [2], atypical localizations such as mediastinum, heart, oesophagus, and diaphragm have also been reported [3]. Its prevalence varies between 1/10.000 and 1/100000 [4]. The incidence is higher in females [4]. In 70% of the patients, there is no normally located thyroid tissue and ectopic thyroid is the only functional thyroid tissue [5]. Therefore, follow-up without treatment is preferred except in cases with obstructive symptoms, bleeding, and suspicion of malignity.

Main findings secondary to the hypertrophy of ectopic thyroid tissue are dysphagia, dysphonia, bleeding, dyspnea, and obstructive sleep apnea [6]. In its management, observation, suppressive treatment, radioactive iodine treatment, and surgery may be preferred.

The first described method in surgical approach is open approaches since it is a region with difficult access. Transoral midline glossotomy, transcervical approach, and transmandibular approach are main surgical approaches, but they may lead morbidity to increase due to the need of lip, tongue, and mandibular splitting or lateral pharyngotomy, requirement of nasogastric tube owing to difficulty in oral intake, the risk of the development of pharyngocutaneous fistula, prolonged hospitalization, and skin scar. Therefore, CO2 laser and electrocauter assisted resection techniques with rigid endoscope and operation microscope and suspension laryngoscopy have been used [7]. However, in these approaches, limited perspective and difficulty in manipulation renders resection more difficult. Transoral robotic surgery decreases the technical difficulties encountered in other transoral approaches in that it has a three-dimensional larger point of view, and manipulation is easier thanks to robotic instruments [8].

In this report, our experience with lingual thyroid resection using transoral robotic surgery is presented.

## 2. Presentation of Case

**Figure 1:**
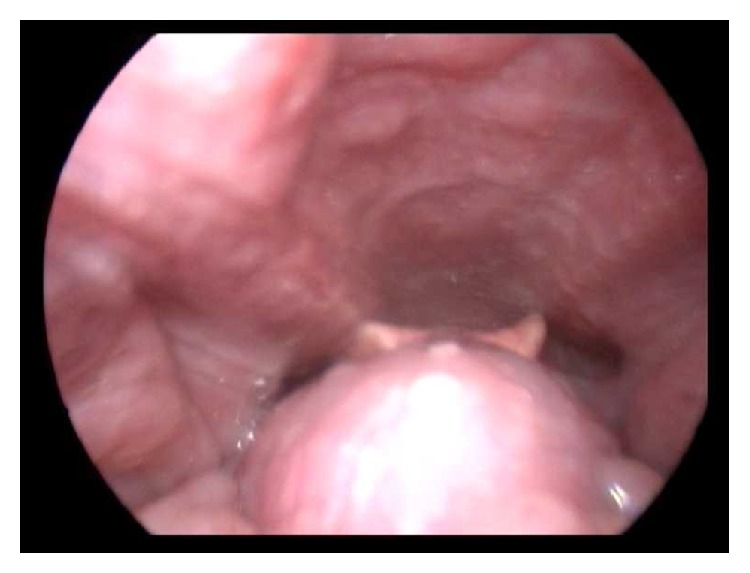
Laryngoscopic appearance of lingual thyroid.

A 49-year-old female patient presented to our clinic with complaints of foreign body feeling in throat, speech disorder, dysphagia, snoring, and sleep apnea, which have become more marked within the last year. In systemic examination, no pathological findings were observed and, in flexible fiber optic laryngoscopic examination, a 2 × 1 cm sized vascularized pink-purple mass lesion at medium consistency was observed (Figure 1). In neck CT, at suprahyoid level, a lesion at the size of 20 × 18 mm, localised at root of tongue, with smooth contours and dense contrast, consistent with ectopic thyroid gland, was observed. Thyroid gland was not observed in its routine position. In thyroid scintigraphy, it was observed that thyroid tissue was not at normal localization and, at the region corresponding to tongue root, activity involvement consistent with ectopic lingual thyroid was present. In laboratory tests, patient was found to be clinically euthyroid. In polysomnography test, AHI was found to be 11.93 and pRDI 15.4. Minimum oxygen saturation was established to be 76.

### 2.1. Surgical Technique

**Figure 2:**
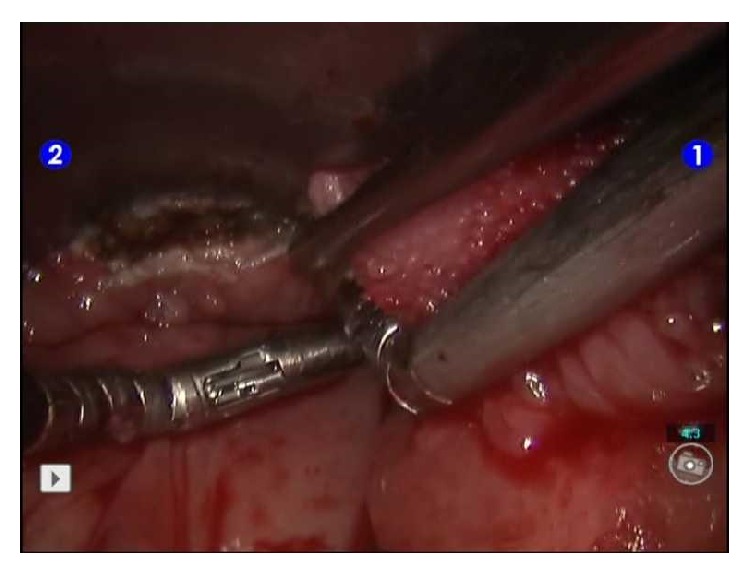
Robotic excision of lingual thyroid tissue.

Operation was carried out under general anesthesia in supine position via nasal intubation. Mass was rendered accessible by traction towards tongue root via 2/0 silk suture. Using Feyh-Kastenbauer-Weinstein-O'Malley (FK-WO) oral retraction, sufficient visualisation was enabled. Operation was carried out by using da Vinci surgical system (Intuitive Surgical, Inc., USA) with 5 mm 3 robotic arms and 0-degree 30-degree high definition 3-dimensional video camera. Resection was carried out by using 5 mm monopolar cauter and Maryland dissector. Incision was started at the anterior of the mass at mucosal level. Posterior traction of the mass facilitates resection by exhibiting the relatively vascular plane between tongue muscles and the mass. Lateral dissection was carried out carefully, taking the course of lingual artery into consideration. Mass was completely separated from the underlying muscular layer with anterior and posterior dissection performed by cauter (Figure 2). Operation was finished after control for postoperative bleeding. No intraoperative or postoperative complications occurred. The duration of operation was 110 minutes. (Robot setting time was 20 min and console time 90 min.)

### 2.2. Postoperative Care

Following operation, patient was extubated and observed. On postoperative day 1, oral soft feeds were initiated. No postoperative bleeding, aspiration, or airway obstruction complications developed. Postoperative L-thyroxine replacement treatment was commenced. In postoperative long term follow-up, obstructive symptoms were reversed.

## 3. Discussion

Although lingual thyroid is reported to be a rare congenital anomaly in the population, in postmortem studies, an incidence as high as 10% has been found [9]. Asymptomatic ectopic thyroid tissue becomes symptomatic in periods of puberty, pregnancy, menopause, inflammation, and stress. Thyroid requirement increasing in these periods leads TSH level to increase and hypertrophy in the thyroid gland [10]. Approach to lingual thyroid tissue may vary according to clinical findings, but it is still debatable.

Although asymptomatic cases can be followed without management for probable complications, in symptomatic cases, there are alternative treatment methods. Even though thyroid suppressive treatment conducted with the administration of exogenous thyroid hormone is a method that can be preferred in patients with minimal symptoms, it is not preferred in that it requires long term treatment. Radioactive iodine treatment and surgery are other treatment alternatives.

As it is located in a region difficult to access, open surgical approaches have been used in surgical treatment [11]. These surgical approaches may lead morbidity rates to increase according to the method used since they require lip, tongue, and mandibular splitting or lateral, suprahyoid pharyngotomy and long operation times and feeding with nasogastric tube, they run the risk of the development of suprahyoid pharyngotomy, hospitalization is prolonged, and there are skin scars. In order to avoid these morbidities, transoral resection techniques including Co2 laser, electrocautery, and hormonic technologies are used. Terris et al. [12] reported that transoral minimal invasive lingual thyroid resection decreased morbidity and duration of hospitalization compared to other open approaches. Coblation assisted lingual thyroid and lingual tonsil resection techniques were used and successful results were reported [13–15]. Leitzbach et al. [14] reported successful results and low complication rates in 108 patients undergoing coblation assisted resection due to tongue root and lingual tonsil hypertrophy. However, transoral resection techniques have limited point of view and lead to difficulty in resection owing to difficulty in manipulation, which restricts their use [7]. Studies have demonstrated that as the size of the mass increases, transoral resectability of the mass decreases. In the resection of tongue root and in lesions over the size of 3 cm, transoral approach is not recommended [12, 16].

Transoral robotic surgery decreases the technical difficulties encountered in other transoral approaches in that it has a three-dimensional larger point of view, and manipulation is easier thanks to robotic instruments, leading to its larger use in oropharyngeal and tongue root operations [8]. Transoral robotic surgery is used in many different regions in surgical otolaryngology [17, 18]. Three robotic arms, 0-degree and 30-degree high definition and 3-dimensional video instruments make it possible to overcome restriction of visualization of regions with difficult access and difficulty of manipulation. Lingual thyroid excision using this method has been described in previous studies [19, 20].

The basic principles of avoiding complications in transoral tongue root resections are careful dissection in accordance with imaging methods and adequate anatomic evaluation in order to prevent lingual artery injury. The evaluation of the relation between the mass and lingual artery is important for avoiding potential complications. Although it has been demonstrated by anatomic studies that lingual artery courses at 1.1.6 cm lateral and 2.2–2.7 cm inferior of foramen caecum, this anatomic relation may be influenced by mass effect [21]. It has been demonstrated that lingual artery may course outside its routine anatomic position [19]. Therefore, evaluation of intralingual neurovascular anatomy is important for avoiding complications.

Major counterindications of this method are limited mouth opening influencing exposition and manipulation and restriction in oropharynx exposition.

## 4. Conclusion

In conclusion, as in other tongue root surgeries, the use of robotic surgery also in lingual thyroid tissue excision decreases morbidity and duration of operation. Studies with larger case series and longer duration of follow-up are required in order to reach a definitive conclusion on this issue.
